# The Effect of Baseline Ovarian Cyst on Pregnancy Outcomes in Ovulation Induction/Intrauterine Insemination Cycles

**DOI:** 10.1089/whr.2023.0124

**Published:** 2024-02-06

**Authors:** Karen Bethel, Rachel Warwar, Suruchi Thakore, Emily G. Hurley

**Affiliations:** ^1^ University of Cincinnati College of Medicine, Cincinnati, Ohio, USA.; ^2^ Division of Reproductive Endocrinology and Infertility, University of Cincinnati, West Chester, Ohio, USA.

**Keywords:** ovulation induction, intrauterine insemination, infertility, ovarian cyst

## Abstract

**Objective:**

To determine the effects of a baseline ovarian cyst on ovulation induction/intrauterine insemination (OI/IUI) cycle outcomes.

**Methods:**

A retrospective cohort analysis of 270 patients and 461 OI/IUI cycles performed between 2011 and 2021 was performed. The exposure variable was a simple appearing ovarian cyst diagnosed at baseline ultrasound measuring ≥10 mm with an estradiol level <75 ng/mL. The primary outcome analyzed was an ultrasound-confirmed intrauterine pregnancy. Secondary outcomes included positive pregnancy test and live birth. Summary data were presented with percentages, mean (standard deviation), or median (interquartile range). Comparisons of dichotomous variables were performed with the chi-square test, and continuous variables were compared using *t*-test. Regression analysis was performed using a general linear model. *p*-Values <0.05 were considered statistically significant.

**Results:**

The clinical pregnancy rate was nominally higher in the group without a cyst present at baseline ultrasound compared with those cycles with a simple cyst present, but the difference was not statistically significant (45/300 [15%] vs. 15/161 [9.3%], risk ratio [RR] 0.63 [0.36, 1.1]). After adjusting for BMI ≥30 and age ≥35, there remained no significant difference in clinical pregnancy rate (adjusted RR 0.65 [0.37, 1.1]).

**Conclusion:**

Given the present data, it is reasonable to proceed with IUI in the case of a baseline simple ovarian cyst. However, this finding may have an impact on clinical pregnancy outcomes in OI/IUI, and further research on the topic is warranted. Although this study was underpowered with fewer cycles than needed to demonstrate a significant difference, the point estimate suggests that the difference in clinical pregnancy rate could be ∼35%.

## Introduction

Ovulation induction with intrauterine insemination (OI/IUI) is a common first-line treatment for both ovulatory dysfunction, which accounts for up to 40% of infertility in women, and unexplained infertility, diagnosed in up to 30% of couples experiencing infertility.^1,2^ The OI cycle protocol consists of medication to stimulate follicular growth, usually achieved by clomiphene citrate (CC) or letrozole (LE), monitored via ultrasound. LE is used commonly in cases of polycystic ovarian syndrome (PCOS) or CC resistance.^3^ Gonadotropins are another alternative in the case of resistance to oral medication.^4^ In most cases, treatment is carried out through at least three cycles of OI/IUI.^5^ Studies have shown that pregnancy rates achieved through OI/IUI are higher than timed intercourse in infertile patients.^6^

Ovarian cysts are common in women undergoing OI/IUI, present in nearly one in five patients.^7^ A simple ovarian cyst is a fluid-filled sac that can transiently form on an ovary during or after ovulation. Other types of cysts can be diagnosed at baseline, including complex and corpus luteal cysts. It is not standard practice to treat baseline simple ovarian cysts.^8^ If a cyst is found to be actively secreting hormone, determined by an elevated estradiol (E2) level, the stimulation cycle is usually canceled, given that the ovaries would have a less than optimal response to the medication. However, if the cyst is not actively producing hormone, the cycle usually proceeds with uncertainty, given the lack of prior research on inactive ovarian cysts and OI/IUI cycles.

Previous studies have established patient selection criteria, including type and duration of infertility, to predict OI/IUI outcomes. In studies screening for significant variables affecting IUI cycle outcomes, sperm count and motility, age, number of follicles, and endometrial thickness were found to impact pregnancy rates, but ovarian cysts were not included in the analysis.^9,10^ Thus, little evidence exists to guide cycle management in the case of an inactive simple ovarian cyst present at baseline ultrasound.

Sufficient counseling about risks, pregnancy rates, and emotional and financial considerations is recommended in consultation before infertility treatments.^5^ Further knowledge and analysis of OI/IUI cycles are important to help patients optimize treatment utility and avoid more costly and invasive treatment modalities such as *in vitro* fertilization. It is necessary to further study OI/IUI cycle characteristics to properly counsel patients regarding the effects of a baseline ovarian cyst on cycle outcomes, and the possible recommendation to defer treatment until the next cycle.

## Materials and Methods

### Study design

This study was a retrospective cohort analysis of OI/IUI cycles performed between 2011 and 2021 at the UC Health Center for Reproductive Health. There were 3712 OI/IUI cycles performed during this time frame, which were reviewed, of which the cycles included in this analysis represent a subset. Study approval was granted by the Institutional Review Board for the Protection of Human Subjects at the University in June 2021. All data were deidentified upon collection and patients were not asked to actively participate.

All data were obtained from Epic electronic medical records and stored in a database created with Research Electronic Data Capture (REDCap) hosted at the University of Cincinnati College of Medicine. REDCap is a secure, web-based application designed to support data capture for research studies, providing (1) an intuitive interface for validated data entry; (2) audit trails for tracking data manipulation and export procedures; (3) automated export procedures for seamless data downloads to common statistical packages; and (4) procedures for importing data from external sources.^11^

### OI/IUI protocol

Patients were between the ages of 22 and 48 and completed OI/IUI using LE or CC with a human chorionic gonadotropin (hCG) trigger. Patients began OI cycles with evaluation between cycle days 2–5 using transvaginal ultrasound to measure endometrial thickness and assess the ovaries. Endometrial measurements were taken as per standard protocol (*ACOG committee opinion 440*). If a simple ovarian cyst was visualized (as defined below), bloodwork for an E2 level was obtained as per the attending physician discretion, and if E2 level was suppressed, the cycle proceeded. Patients proceeding with OI/IUI were treated with oral LE or CC daily starting on days 3–5 of their menstrual cycle, for a total of 5–10 days. LE dose ranged between 2.5 and 10 mg/day, and CC dose ranged from 50 to 200 mg/day.

Doses were chosen based on patient age, weight, duration of infertility, and previous unsuccessful OI cycles. The hCG trigger was administered in a single 250 mg subcutaneous injection to stimulate ovulation once follicles reached the appropriate size, ∼20–21 mm in average diameter. Patients underwent IUI 24 or 36 hours after hCG trigger was administered.

### Quantitative variables

The exposure variable was a simple appearing ovarian cyst diagnosed at baseline ultrasound measuring ≥10 mm with an E2 level <75 ng/mL. OI/IUI cycles with an active baseline cyst (E2 level ≥75 ng/mL) were excluded. Noncyst cycles were chosen at random. There were no means of matching for variables potentially affecting the cycles. Exclusion criteria included cycles with documented paratubal cyst, echogenic cyst, hemorrhagic cyst, dermoid cyst, corpus luteal cyst, or endometrioma. Cycles using gonadotropins in addition to or *in lieu* of CC or LE for ovarian stimulation before hCG trigger were excluded. Patients with history of infertility secondary to cancer, chemotherapy, or radiation were also excluded.

The primary outcome observed was clinical pregnancy, defined as ultrasound-confirmed intrauterine pregnancy (IUP). Secondary outcomes observed were biochemical pregnancy, defined as a positive urine pregnancy test (UPT) or quantitative hCG >5 mIU/mL, and live birth, defined as delivery after 24 weeks of gestation.

### Data collection

Demographic characteristics of patients and cycle details were collected. Diagnoses included the following: PCOS, male factor, tubal factor, diminished ovarian reserve, endometriosis, and unexplained infertility. Patients with amenorrhea, anovulation, cervical stenosis, Asherman syndrome, fibroid, or vaginismus were placed in a diagnosis category labeled “other” for analysis.

Cycle characteristics included the following: infertility diagnosis (as above), medication for OI (LE or CC), dosage, location and size of follicular growth, and endometrial thickness before hCG trigger. Cycles with a simple appearing ovarian cyst(s) diagnosed at baseline ultrasound also included number of cysts and E2 level. If medication redosing was required, the higher dose was recorded. If a cyst or follicle was measured in dimensions, the average diameter was recorded. When ultrasound was not performed on the day of hCG trigger, a predetermined value of 2 mm was added to each follicle per day between ultrasound and day of trigger. Likewise, 1 mm was added to the endometrial thickness per day between ultrasound and day of trigger. If no follow-up was reported in the medical records, the outcome was assumed to be a negative UPT.

### Statistical analysis

A statistical power analysis was performed to determine the sample size needed to detect a 50% difference in primary outcome between exposed and unexposed groups. This proposed sample size was 280 control cycles and 280 cycles with cyst at baseline. Statistical analysis of descriptive characteristics and outcomes was performed using STATA Statistics and Data Science Basic Edition 17.0.^12^ Dichotomous variables were analyzed by Pearson's chi-square tests, and continuous variables were analyzed by two-sample unpaired *t*-test. A *p*-value <0.05 was considered statistically significant. Regression analysis was performed using a general linear model to identify prognostic variables for an adjusted risk ratio (aRR) to reduce potential confounding of age ≥35 and body mass index (BMI) ≥30.

## Results

A total of 270 patients and 461 OI/IUI cycles were included in the analysis, of which 300 were control cycles and 161 were cycles with at least 1 baseline simple cyst. Goal sample size was not achieved due to the unexpected rarity of simple cysts in isolation, with low E2 levels.

Patient and OI/IUI cycle characteristics are reported in Table 1. On average, patients with ovarian cyst(s) were more likely to be older (*p* < 0.0001), and have a higher BMI (*p* = 0.0002), Anti-Mullerian hormone level (*p* = 0.0001), and gravidity (*p* = 0.0008). The following diagnoses were associated with a higher incidence of baseline cyst: diminished ovarian reserve (*p* = 0.007) and “other” (*p* = 0.032). PCOS was associated with a lower incidence of baseline cyst (*p* = 0.004) (Table 1).

**Table 1.** Patient Demographics and Cycle Characteristics in Ovulation Induction/Intrauterine Insemination Cycles With and Without Baseline Ovarian Cyst(s)

	No cyst at baseline ultrasound ***n*** = 300	Simple cyst(s) present at baseline ultrasound measuring 10–55 mm with E2 level <75 ng/mL ***n*** = 161	***p***
Age	31.8 (4.5)	34.0 (4.5)	<0.0001^b^
BMI	27.6 (7.2)	30.4 (8.2)	0.0002^b^
Gravidity	0 (0, 1)	0 (0, 2)	0.0008^a,b^
Parity	0 (0, 0)	0 (0, 0)	0.081^a^
Diagnosis			
Polycystic ovary syndrome	112/300 (37.3%)	39/161 (24.2%)	0.004^b^
Male factor	132/300 (44.0%)	60/161 (37.3%)	0.16
Tubal factor	14/300 (4.7%)	9/161 (5.6%)	0.66
Diminished ovarian reserve	28/300 (9.3%)	29/161 (18.0%)	0.007^b^
Endometriosis	18/300 (6.0%)	8/161 (5.0%)	0.65
Unexplained Infertility	56/300 (18.7%)	37/161 (23.0%)	0.27
Other	21/300 (7.0%)	21/161 (13.0%)	0.032^b^
≥2 diagnoses	74/300 (24.7%)	40/161 (24.8%)	0.97
Anti-Mullerian hormone level (ng/mL)	4.6 (4.4)	3.0 (4.2)	0.0001^b^
Medication and dose (mg)			
Letrozole	184/300 (61.3%)	92/161 (57.1%)	0.38
2.5	4/184 (2.2%)	0/92 (0.0%)	0.002^b^
5	90/184 (48.9%)	26/92 (28.3%)	
7.5	76/184 (41.3%)	53/92 (57.6%)	
10	14/184 (7.6%)	13/92 (14.1%)	
Clomid	116/300 (38.7%)	69/161 (42.9%)	0.38
50	7/116 (6.0%)	2/69 (2.9%)	0.56
100	75/116 (64.7%)	44/69 (63.8%)	
150	33/116 (28.5%)	21/69 (30.4%)	
200	1/116 (0.86%)	2/69 (1.6%)	
Endometrial lining at trigger (mm)	9.3 (1.9)	8.9 (1.9)	0.024^a,b^
No. of mature follicles at trigger			
0	7/300 (2.3%)	1/161 (0.62%)	0.44
1	159/300 (53.0%)	79/161 (49.4%)	
2	107/300 (35.7%)	63/161 (39.4%)	
≥3	27/300 (9.0%)	17/161 (10.6%)	
Size of leading follicle at trigger	21.0 (2.1)	21.8 (2.5)	0.0002^a,b^
Location of follicular growth			
Left	115/300 (38.3%)	60/161 (37.5%)	0.98
Right	112/300 (37.3%)	60/161 (37.5%)	
Bilateral	73/300 (24.3%)	40/161 (25.0%)	
Cycle day of trigger	12 (11, 13)	11.5 (11, 13)	0.41^a^

^a^ Median and interquartile range reported for continuous variables of non-normal distribution.

^b^ Statistically significant *p*-value <0.05.

BMI, body mass index; E2, estradiol.

Table 2 summarizes characteristics of baseline simple ovarian cyst(s) present in the cyst group. With a cyst present, follicular growth occurred on the opposite ovary in 64% of cases (Table 2).

**Table 2.** Characteristics of Simple Appearing Ovarian Cysts Diagnosed at Baseline Ultrasound Measuring 10–55 mm with Estradiol Level <75 ng/mL (*n* = 161)

No. of cysts present at baseline ultrasound, mean	1.1
No. of cysts present at baseline ultrasound, range	(1,3)
Size of largest cyst (mm), mean	20.2
No. of cycles with cyst <15 mm	48/161 (29.8%)
No. of cycles with cyst 15–20 mm	57/161 (35.4%)
No. of cycles with cyst >20 mm	56/161 (34.8%)
Location of cyst(s)	
Left	74/161 (46.0%)
Right	79/161 (49.1%)
Bilateral	8/161 (4.7%)
Cyst location with respect to follicular growth location	
Same side	58/161 (36.0%)
Opposite side	103/161 (64.0%)
E2 level (ng/mL), mean	43.6
E2 level (ng/mL), range	(9.5, 74)

The incidence of a positive pregnancy test was nominally higher in the group without a cyst present at baseline ultrasound compared with cycles with a cyst present, 53/300 (17.7%) versus 17/161 (10.5%), but this difference was not significant, RR = 0.61 (0.36, 1.01). Of the cycles without cysts that resulted in a positive pregnancy test, 8 were found to be biochemical pregnancies and 43 were clinical IUPs. Of the cycles with cysts that resulted in pregnancy, 2 were found to be biochemical pregnancies and 15 were clinical IUPs. There were no ectopic pregnancies in the study population (Table 3).

**Table 3.** Ovulation Induction/Intrauterine Insemination Cycle Outcomes With and Without Baseline Ovarian Cyst

	No cyst present at baseline ultrasound ***n*** = 300	Simple cyst(s) present at baseline ultrasound measuring 10–55 mm with E2 level <75 ng/mL ***n*** = 161	RR (95% CI)	aRR (95% CI) Adjusted for advanced maternal age (≥35 years) and high BMI (≥30)
Pregnancy	53/300 (17.7%)	17/161 (10.5%)	0.61 (0.36, 1.01)	0.62 (0.37, 1.0)
Pregnancy of unknown location	8/53 (15.1%)	2/17 (11.8%)		
Ectopic pregnancy	0/53 (0%)	0/17 (0%)		
Clinical pregnancy	45/300 (15%)	15/161 (9.3%)	0.63 (0.36, 1.1)	0.65 (0.37, 1.1)
Live birth^a^	35/300 (11.7%)	5/161 (3.1%)	0.61 (0.36, 1.0)	0.37 (0.06, 2.2)

^a^ Live birth data missing for 7 (46.7%) cyst cycles and 7 (15.6%) noncyst cycles.

aRR, adjusted RR; RR, risk ratio.

Clinical IUP was achieved in 15% of the noncyst group versus 9.3% of the cyst group, RR = 0.63 (0.36, 1.1). These results were relatively unchanged after adjusting for age ≥35 and BMI ≥30 (aRR = 0.65) (Table 3).

**FIG. 1.**
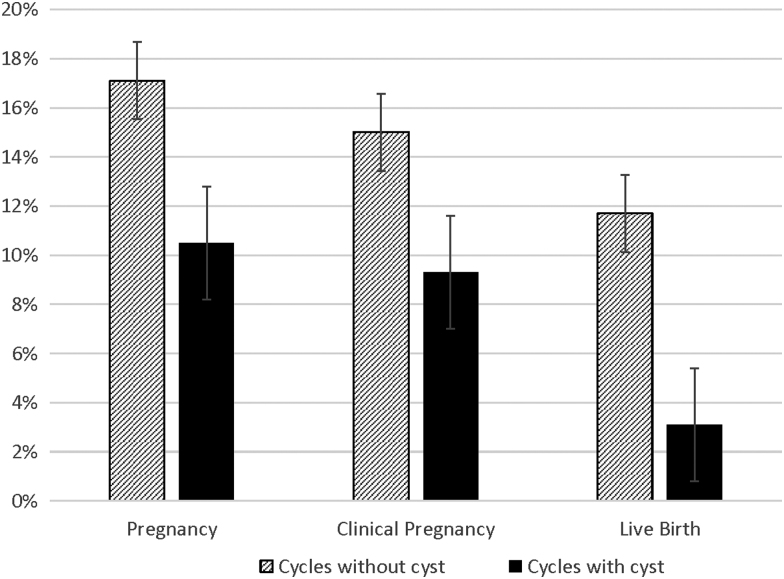
Ovulation induction/intrauterine insemination cycles with baseline ovarian cyst are less likely to result in pregnancy, clinical pregnancy, and live birth.

The noncyst group had 35/300 (11.7%) live births compared with the cyst group with 5/161 (3.1%) live births, RR = 0.61 (0.36, 1.0). These outcome data were missing for 7 (15.6%) pregnancies in the noncyst group and for 7 (46.7%) pregnancies with a baseline cyst (Fig. 1).

There was no statistically significant association between baseline cyst and unexplained infertility diagnosis, and therefore, a subgroup analysis in cycles with unexplained infertility was assessed. In these cycles, 56 without cysts and 37 with cysts, a baseline cyst did not have an effect on clinical pregnancy outcomes, RR = 0.95 (0.37, 2.7) and aRR = 0.99 (0.35, 2.8) (Table 3).

## Discussion

In this study, we evaluated the effect of the presence of an inactive simple ovarian cyst at baseline ultrasound on OI/IUI outcomes. No significant difference in outcomes, including a positive pregnancy test, clinical pregnancy, or live birth, was found between the cycles with and without baseline simple cyst(s). Further emphasizing the necessity of investigating the effects of ovarian cyst on OI/IUI, our patient demographics analysis confirms that an ovarian cyst can be present regardless of diagnosis.

There is limited available literature evaluating the effects of an inactive simple ovarian cyst on OI/IUI cycle outcomes. One study found that baseline ovarian cysts reduced spontaneous ovulation in patients treated with CC. This study was not powered to compare pregnancy rates; however, the pregnancy rates were not statistically different. In addition, this study did not evaluate if the cyst was actively producing hormone at the beginning of the cycle.^5^ Another study comparing the use of CC versus placebo in those with functional ovarian cysts at baseline found that cysts significantly reduced ovulatory events in patients treated with CC, without a difference in pregnancy rates.^13^ Neither of these studies evaluated patients undergoing IUI, and no hCG trigger was used.

A randomized controlled trial of 60 patients with ovarian cysts undergoing OI found that CC increased ovulation and pregnancy rates.^14^ Again, this study did not specifically investigate IUI cycles and there was no control group without cysts. A cross-sectional analysis of factors influencing pregnancy rate in IUI cycles with hCG stimulation found increased pregnancy rate with three preovulatory follicles, infertility duration <3 years, and a dose–response trend in total motile sperm count and pregnancy outcome, yet there was no analysis of ovarian cyst.^15^ The lack of research evaluating the effects of inavtive baseline ovarian cysts in fertility treatment cycles using hCG trigger makes it difficult to counsel patients seeking OI/IUI with inactive simple cysts detected at their baseline ultrasound.

This study was underpowered with fewer cycles than needed to demonstrate a significant difference due to the unexpected rarity of finding simple cyst(s) in isolation, with low E2 levels. For example, simple cysts were often found with a complex cyst or with an elevated E2 level, which would exclude the cycle from analysis. However, we still believe that isolating the specific exposure is beneficial for analysis. Additional studies using a complex cyst as the exposure could further increase the ability to counsel patients presenting with more than one type of cyst at baseline ultrasound.

Although not statistically significant, the point estimate suggests that the difference could be ∼35% and further data are needed. Going forward, an estimated sample size of at least 1232 cycles (616 in each group) could provide adequate power to evaluate this potential difference based on a control incidence of 0.15 and a beta-level of 0.2.

This analysis is limited by the differences in baseline patient and cycle characteristics of the two groups shown in Table 1. Of these differences in baseline characteristics, age and BMI were chosen for statistical adjustment, as the present data set was not large enough to support the use of more than two covariates. In addition, organized means for random sampling and matching for factors known to affect OI/IUI could reduce selection bias in future studies.

Limitations due to the retrospective nature of this study are evident, including missing data. For example, an E2 level was not obtained for several cysts of smaller size, but it was assumed that they were inactive, as the cycles proceeded to IUI. Live birth outcome data were missing in some cases that were too recent to be seen through to term, as well as in patients transferred to other institutions without available medical records. When ultrasound was not performed on the exact day of hCG trigger, measurements had to be estimated by adding predetermined values.

The results of the subgroup analysis of unexplained infertility cycles, which showed no difference in outcomes between cycles with cysts and those without, suggest that differences in pregnancy outcomes may be due to unknown underlying factors affecting fertility. It also suggests that patients with unexplained infertility may need to be counseled differently than those with a specific diagnosis such as PCOS. This study was not powered to evaluate this subgroup, but this reinforces the need for additional data to further investigate outcomes associated with specific diagnoses.

In conclusion, this study yielded no significant difference in pregnancy outcomes for OI/IUI cycles with and without an inactive simple ovarian cyst at baseline ultrasound. Thus, it is reasonable to proceed with an OI/IUI cycle in this circumstance. Nonetheless, these results are important in guiding future studies given a potential negative effect of cysts on pregnancy rates. As a 35% decrease in the incidence of IUP would be considered clinically significant, subsequent studies with additional data are needed to further evaluate the impact of ovarian cysts on OI/IUI outcomes.

## Data Sharing Statement

The data associated with this study are stored in a REDCap Database, available from the corresponding author upon request.

## Abbreviations Used

aRR: adjusted risk ratio
BMI: body mass index
CC: clomiphene citrate
E2: estradiol
hCG: human chorionic gonadotropin
IUP: intrauterine pregnancy
LE: letrozole
OI/IUI: ovulation induction/intrauterine insemination
PCOS: polycystic ovarian syndrome
REDCap: Research Electronic Data Capture
RR: risk ratio
UPT: urine pregnancy test
